# Cyclic strain upregulates VEGF and attenuates proliferation of vascular smooth muscle cells

**DOI:** 10.1186/2045-824X-3-21

**Published:** 2011-09-19

**Authors:** Joseph F Schad, Kate R Meltzer, Michael R Hicks, David S Beutler, Thanh V Cao, Paul R Standley

**Affiliations:** 1 Department of Basic Medical Sciences, University of Arizona - Phoenix, AZ, USA; 2 Department of Biomedical Sciences, Midwestern University - Glendale, AZ, USA; 3 Department of Molecular and Cell Biology, Arizona State University - Tempe, AZ, USA

**Keywords:** blood vessels, cell proliferation, biomechanical strain, VEGF, vascular smooth muscle, cytokines, nitric oxide

## Abstract

**Objective:**

Vascular smooth muscle cell (VSMC) hypertrophy and proliferation occur in response to strain-induced local and systemic inflammatory cytokines and growth factors which may contribute to hypertension, atherosclerosis, and restenosis. We hypothesize VSMC strain, modeling normotensive arterial pressure waveforms in vitro, results in attenuated proliferative and increased hypertrophic responses 48 hrs post-strain.

**Methods:**

Using Flexcell Bioflex Systems we determined the morphological, hyperplastic and hypertrophic responses of non-strained and biomechanically strained cultured rat A7R5 VSMC. We measured secretion of nitric oxide, key cytokine/growth factors and intracellular mediators involved in VSMC proliferation via fluorescence spectroscopy and protein microarrays. We also investigated the potential roles of VEGF on VSMC strain-induced proliferation.

**Results:**

Protein microarrays revealed significant increases in VEGF secretion in response to 18 hours mechanical strain, a result that ELISA data corroborated. Apoptosis-inducing nitric oxide (NO) levels also increased 43% 48 hrs post-strain. Non-strained cells incubated with exogenous VEGF did not reproduce the antimitogenic effect. However, anti-VEGF reversed the antimitogenic effect of mechanical strain. Antibody microarrays of strained VSMC lysates revealed MEK1, MEK2, phospo-MEK1^T385, T291, T298^, phospho-Erk1/2^T202+Y204/T185+T187^, and PKC isoforms expression were universally increased, suggesting a proliferative/inflammatory signaling state. Conversely, VSMC strain decreased expression levels of Cdk1, Cdk2, Cdk4, and Cdk6 by 25-50% suggesting a partially inhibited proliferative signaling cascade.

**Conclusions:**

Subjecting VSMC to cyclic biomechanical strain in vitro promotes cell hypertrophy while attenuating cellular proliferation. We also report an upregulation of MEK and ERK activation suggestive of a proliferative phenotype. Hhowever, the proliferative response appears to be aborogated by enhanced antimitogenic cytokine VEGF, NO secretion and downregulation of Cdk expression. Although exogenous VEGF alone is not sufficient to promote the quiescent VSMC phenotype, we provide evidence suggesting that strain is a necessary component to induce VSMC response to the antimitogenic effects of VEGF. Taken together these data indicate that VEGF plays a critical role in mechanical strain-induced VSMC proliferation and vessel wall remodeling. Whether VEGF and/or NO inhibit signaling distal to Erk 1/2 is currently under investigation.

## Introduction

Cyclic strain (CS) induced by changes in blood pressure can regulate vascular remodeling, proliferation, apoptosis, cell phenotypic changes, and secretion of extracellular matrix proteins and cytokines [1]. Pathological states such as hypertension, atherosclerosis, and restenosis lead to further changes in vascular remodeling [1]. Vascular smooth muscle cells (VSMC) are prevalent in vessel walls and are mechanotransducers of strain and shear stress [2]. In vitro biophysical strain models that mimic arterial pressure waveforms have provided important mechanistic explanations about VSMC responses [2,3]. For instance, under normotensive conditions, hemodynamic forces strain large arteries up to 10% (termed "physiological" strain; [4]), which shifts VSMC from a synthetic/secretory phenotype in static culture to a non-proliferative contractile phenotype [5-7]. Further elevation of strain magnitude (15 - 30%) [4] shifts cells to a contractile proliferative phenotype [7].

Mechanotransduction cascades have been implicated in the regulation of the VSMC proliferative response [2] including VSMC-derived cytokines such as IGF-1 and PDGF [8-10]. Vascular endothelial growth factor (VEGF) is also an important repressor of VSMC proliferation [11,12], and is upregulated in aorta from hypertensive rats [13] and in strained VSMC cultures [14]. VSMC calcium homeostasis also plays a pivotal role in strain-induced VSMC proliferation which is dependent upon both stretch activated calcium channels (SACC) and voltage operated calcium channels [15]. Thus the proliferative effects of biomechanical strain on VSMC are dependent upon the complex interplay of the intracellular signaling cascades involved in proliferation and progression of the cell cycle.

The current study investigates the antimitogenic effects of a 10% CS on VSMC. We assessed strain-regulation of autocrine cytokine and growth factor secretion and hypothesized that VEGF plays an important role in mediating strain-induced antimitogenic behavior. We also examined the potential role of SACCs in mediating this antiproliferative effect and the expression and phosphorylation of the mechanosensitive ERK1/2 signaling cascade, PKC isoforms, and Cdk isoforms.

## Materials and methods

### Vascular Smooth Muscle Cell Cultures

A7r5 rat thoracic aortas VSMC were purchased from American Type Culture Collection (Rockville, MD). Cells were cultured in Dulbecco's modified Eagles Medium (DMEM) supplemented with 9% fetal bovine serum (FBS) and 1% penicillin-streptomycin at 37°C, 5% CO_2_, and 100% humidity. Growth medium (GM) was replaced every other day, and upon 80% confluency cells were passed at a ratio of 1 to 4 (acquired in 4 to 5 days). All experiments utilized passage-matched VSMC between passage 2 and 10.

### In Vitro Strain Apparatus

The Flexercell FX-4000 Tension Plus (Flexcell International Corp, Hillsborough, NC) is a computer-based system which utilizes vacuum suction to strain cells seeded on flexible collagen I-coated electrometric membranes. The deformation of the elastomeric membrane causes the adherent cells to similarly deform. The strain profile was created by programming the magnitude, duration, and frequency of negative pressure to match the desired model. Strain on the cells utilizing this apparatus has been empirically determined to be heterobiaxial [16].

### Strain Profile

**Figure 1:**
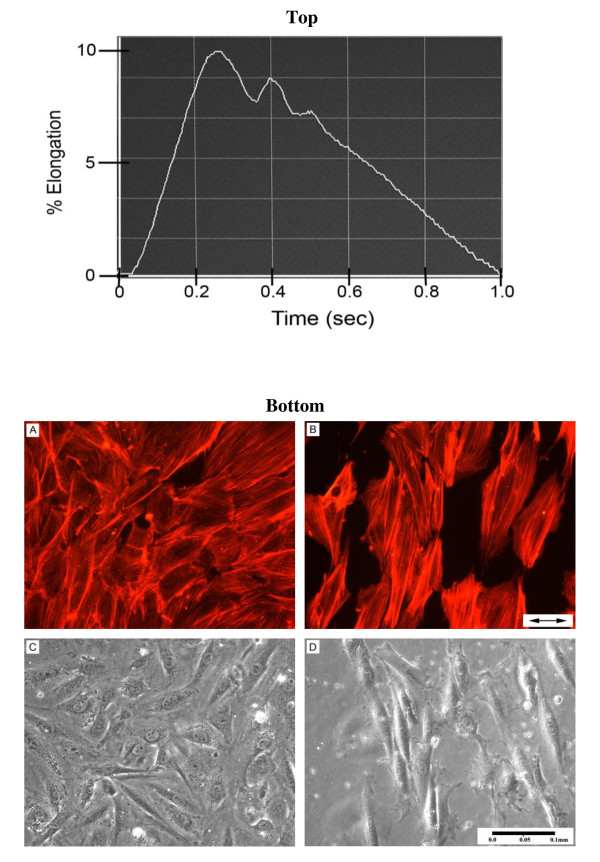
**Aortic Pressure Waveform Regulates VSMC Alignment**: (Top Panel) Strain profile used in this study. Control cells were not strained. (Bottom Panel) Control VSMC (A, C) and VSMC strained cyclically for 48 hours at 1 Hz, 10% (B, D). Representative wells in each experimental group were fluorometrically stained for assessment of intracellular actin (A, B). Arrows in panel B indicate the direction of the dominant strain vector.

Cells were seeded (65,000 cells/well) onto 6-well collagen I-coated Bioflex plates (Flexcell International Corp, Hillsborough, NC). Once the cells were ~50-60% confluent (~24 hours after seeding), the GM was replaced with serum-free medium for 24 hours to induce quiescence, and on the day of the experiment the medium was replaced with fresh GM. VSMC were then cyclically strained for 1, 6, 18 or 48 hours. The strain paradigm modeled an aortic pressure waveform and included a modeled dicrotic notch (Figure 1 top). Control cells were grown in the identical environment, but were not subjected to strain.

### Cell viability, growth assessments, and morphology

A subset of control and strained cells was lysed and cell lysates were used for dsDNA (Invitrogen Corporation; Eugene, OR) and protein quantification (Pierce Chemical Co., Rockford, IL). The ratio of protein to dsDNA was calculated as one proxy of cellular hypertrophy. Phase contrast microscopy at 48 hours was used to quantify cell viability, cell number, cell area and perimeter, and morphologic changes. Actin architecture changes were assessed using rhodamine-conjugated phalloidin as previously described [17]. Photomicrographs were digitally captured using an IX71 Olympus inverted fluorescent microscope and DP71 camera (Olympus America Inc, Melville, NY) and cell area and perimeter measure obtained via Image J v1.37 (National Institute of Health; http://rsb.info.nih.gov/ij/). Cells were randomly selected from four representative experiments and at least four cells from each photographic quadrant were measured in blinded fashion (control: n = 17; strain: n = 16). Cells per high powered field (cells/hpf) were calculated from digital images of control (n = 24) and strained (n = 27) cells from four experiments.

### Cytokine Detection

Conditioned media samples obtained from six control and strained samples were collected at the end of 48 hours and frozen at -80°C until assayed. Array membranes were prepared and percent change from control (%CFC) was determined by methods described previously [18]. Further assessment of VEGF was done using a rat enzyme-linked immunosorbent assay (ELISA) kit (Raybiotech Inc., Norcross, GA). VSMC strain-derived NO was quantified using a fluorometric assay kit, (Cayman Chemical Company, Ann Arbor, MI). All data were normalized to cell proliferation data.

### Exogenous VEGF Experiments

In a subset of experiments, quiescent medium was replaced with 100 μl of GM containing rat VEGF (R&D Systems, Minneapolis, MN) concentrations of 0, 0.003, 0.03, 0.3, 3, and 30 ng/ml. Cells were then incubated for an additional 18 or 48 hours and proliferation rates at these two time points were measured using the CellTiter 96^® ^Aqueous One Solution Cell Proliferation Assay (MTS). Neither control nor VEGF-treated VSMC were strained in this experimental series. In a separate set of experiments, control and strained VSMC were treated with 0.4 μg/ml of anti-VEGF (R&D Systems, Minneapolis, MN) and assess for cell proliferation by method previously described.

### Effects of Extracellular Calcium and Gadolinium

In a subset of experiments, serum-free medium was replaced with one of the following growth media containing 9% FBS: 1) Hyclone custom calcium-free medium (CFM; Thermo Scientific Hyclone AdvanceStem, Logan, UT) with 0.0018 moles/L CaCl_2_, 2) CFM with 0.0018 moles/L NaCl (osmotic control), and 3.) CFM with 0.0018 moles/L CaCl_2 _and 30 μM of GdCl_3_. Cells were then strained or left static for 48 hours.

### Intracellular Protein Microarrays

Antibody microarray services from Kinexus Bioinformatics Corporation (Vancouver, BC, Canada) detected the expression and phosphorylation states of 608 cell signaling proteins (in duplicate) after 48 hours strain from 10 culture wells of control and strained VSMC. Samples were prepared per Kinexus protocol; five wells were collectively pooled for each assay based upon the media and protein requirements for detection. Protein spotting and hybridization onto the array, scanning, imaging, and quantitative analysis of the enhanced chemiluminescence signal of the signaling proteins was performed (in a blinded fashion) by Kinexus Bioinformatics Corporation. Only proteins exhibiting a %CFC of ± 25% were considered for further analysis.

### Replicates and statistical analysis

Experiments were completed a minimum of three times and had three or more replicate wells per experiment, except for the anti-VEGF and calcium/gadolinium experiments which was completed over two experiments with three or more replicate wells. Kinexus antibody microarray experiments consisted of ten pooled replicate wells and the assay was done in duplicate. All data are expressed as mean ± SEM and means were compared by two-tailed unpaired t-tests or one-sample two-tailed t-tests where appropriate. We tested for polyploidy (dsDNA:cell) by calculating the variance of ratios [19] between the mean ± SEM dsDNA and the mean ± SEM cells per high powered field for both strained and non strained VSMC; p < 0.05 were determined to be significantly different. All data were analyzed with Microsoft Excel (Microsoft Corporation) and Prism 4.03 (GraphPad Software, Inc., San Diego, California).

## Results

**Figure 2:**
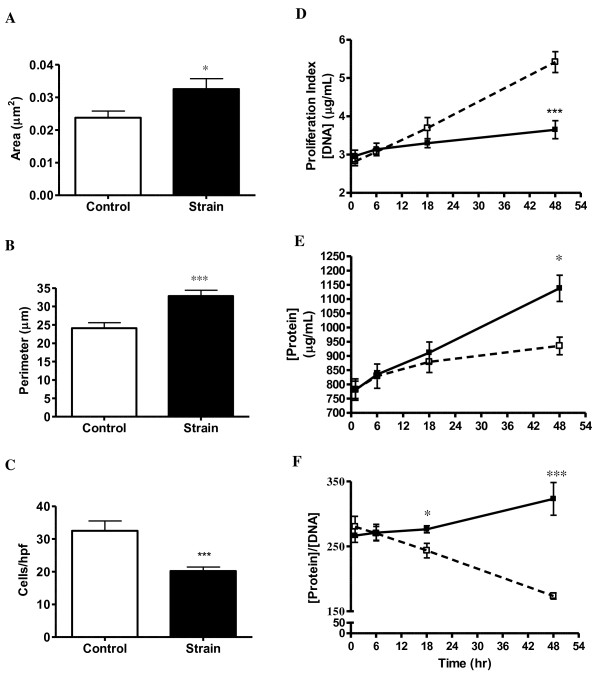
**Strain Regulation of VSMC Hyperplasia and Hypertrophy**: VSMC area (A), perimeter (B), and cells per high power field (hpf; panel C) data from control (N = 17) and strained (N = 16) cells. Proliferation index (D), cellular protein (E), and protein/DNA ratio (F) data for control (open symbols) and strained (closed symbols) VSMC. N = 6 to 9 per group; *p < 0.05; ***p < 0.001 vs. control at the same time point.

Intracellular actin and long axes of strain-induced VSMC cytoskeleton aligned nearly perpendicularly to the dominant strain vector (Figure 1; bottom panels B and D). Non-strained VSMC displayed no such alignment characteristics (Figure 1; bottom panels A and C). Strained VSMC also exhibited decreased proliferation and increased cell size versus control. Strained cells had an average of 37% greater area (p < 0.05), 36% larger perimeter (p < 0.001) and 38% fewer cells/hpf (p < 0.001) when compared to control after 48 hours of CS (Figure 2A-C). Double-stranded DNA concentration (Figure 2D) was also reduced by 33% in strained VSMC (p < 0.001). To confirm this dsDNA reduction was not a function of cellular polyploidy as is common among hypertensive VSMC, we calculated the concentration dsDNA:cells/hpf ratio of strained vs. control (0.181 ± 0.016 μg DNA/cell vs. 0.167 ± 0.056 μg DNA/cell) and determined there was no significant difference (p > 0.05). Strained VSMC exhibited an 18% increase in total cell protein (p < 0.01; Figure 2E) and a 12% higher protein to DNA ratio (proxy of hypertrophy) at 18 hours (p < 0.05) and 46% at 48 hours (p < 0.001). We also calculated the protein concentration:cells/hpf ratio of strained vs. control (56.4 ± 4.0 μg protein/cell vs. 28.8 ± 2.8; μg protein/cell p < 0.05) and in this case we found there to be a significant difference.

**Figure 3:**
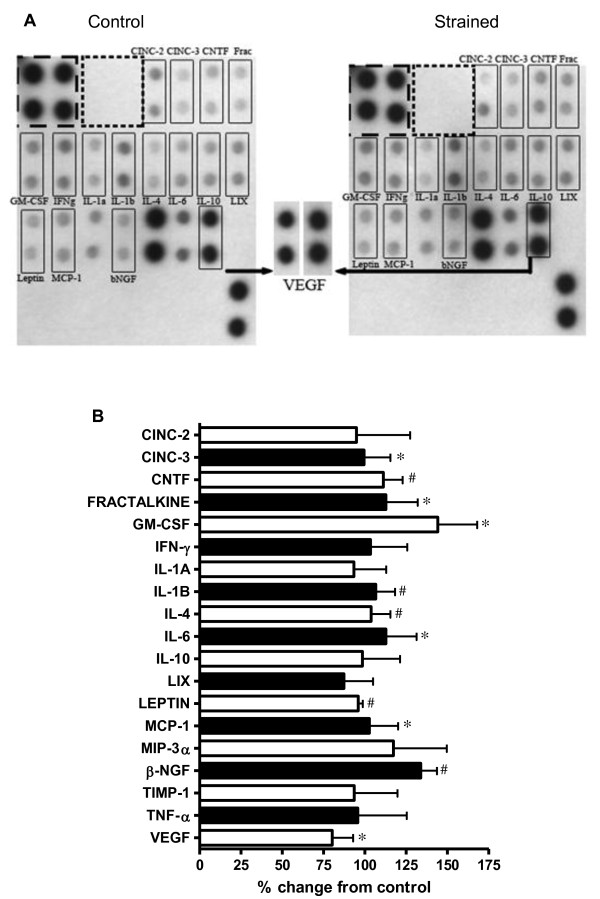
**Strain Regulation of VSMC Cytokine Secretion**: Changes in cytokine levels in conditioned media from control and strained VSMC. (A) Representative matched protein microarrays from control (left) and strained (right) groups. VEGF signals have been enlarged for clarity. (B) Summary data for six protein microarrays showing percent change from control in cytokine levels *p < 0.05 vs. %CFC > 25%; ^#^p < 0.05 vs. %CFC > 50%. All data are corrected on a per-cell basis. *CINC-2 (cytokine induced neutrophil chemoattractant-2), CINC-3 (cytokine induced neutrophil chemoattractant-3), CNTF (ciliary neuronotrophic factor), GM-CSF (granulocyte-macrophage colony stimulating factor), IFN-γ (interferon-γ), LIX (lipopolysaccharide-induced CXC chemokine), MCP-1 (monocyte chemotactic protein-1), MIP-3α (macrophage inflammatory protein-3-alpha), β-NGF (beta-nerve growth factor), TIMP-1 *(*tissue inhibitor of metalloproteinases), TNF-α (tumor necrosis factor α), and VEGF (Vascular Endothelial Growth Factor)*.

**Figure 4:**
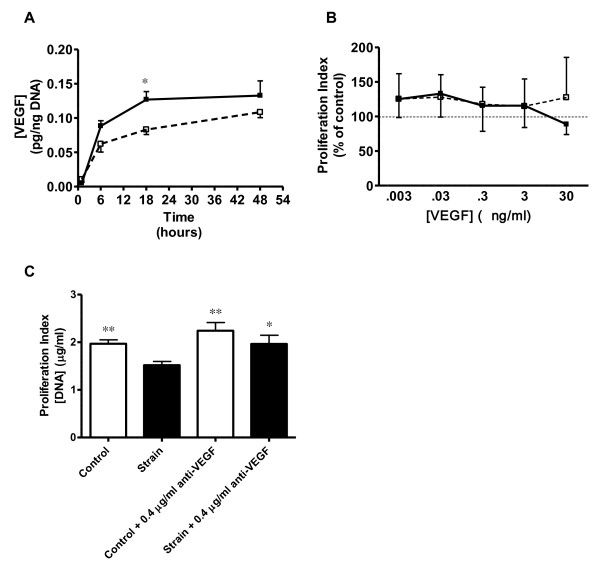
**Effects of Strain on VEGF Secretion and VEGF Blockade on VSMC Proliferation**: (A) Temporal changes in VEGF secretion from control (open symbols) and strained VSMC. N = 5 per group; * p ≤ 0.05 vs. control at same time point. (B) Lack of significant effects of 18 hours (closed symbols) and 48 hours (open symbols) of exogenous VEGF treatment on proliferation in unstrained VSMC (N = 9). (C) Effects of strain (closed bars) and anti-VEGF treatment on VSMC proliferation. N = 6; *p < 0.05 vs strain only group; **p < 0.01 vs strain only group.

Compared to control cells at 48 hours, strained VSMC exhibited significant changes in the secretion of CINC-3, CNTF, Fractalkine, GM-CSF, IL-1β, IL-4, IL-6, Leptin, MCP-1, β-NGF and VEGF (p < 0.05 for all; Figure 3). Further analysis of VEGF secretion via ELISA showed a 57% (p < 0.05) increase after 18 hours strain and 22% increase (p > 0.05) after 48 hours strain when compared to their respective controls (Figure 4A). VSMC also secreted 65% more NO after 48 hours of CS compared to control (p < 0.05; Table 1).

**Table 1:** Temporal changes in NO secretion from control and strained VSMC.

	Nitric Oxide (nM/ng DNA)		Proliferation Index	
		
Time (hours)	Control	Strain	Treatment	[DNA]/(ug/ml)
1	0.13 ± 0.013	0.13 ± 0.014	Control + Ca	6.28 ± 0.69
6	0.09 ± 0.025	0.10 ± 0.016	Strain + Ca	2.98 ± 0.66 **
18	0.12 ± 0.016	0.14 ± 0.013	Strain - Ca	3.30 ± 0.70 *
48	0.07 ± 0.009	0.12 ± 0.013^#^	Strain + Ca + GdCl_3_	2.62 ± 0.37 ***

N = 5 per group; ^#^p ≤ 0.05 vs. control at same time point. The effects of strain, calcium-free media and GdCl_3 _on VSMC proliferation. N = 6; *p < 0.05 vs. Control + Ca, **p < 0.01 vs. Control + Ca, ***p < 0.001 vs. Control + Ca; all values are expressed as mean ± SEM.

In the absence of strain, 18 and 48 hours incubation with various concentrations of exogenous VEGF did not affect VSMC proliferation when compared to control (Figure 4B). However, the addition of 0.4 μg/ml anti-VEGF (100 times neutralizing dose, 50%) to VSMC prior to a CS regimen caused a 29% increase (p < 0.01) in proliferation accompanied by 26% decrease in protein expression (p < 0.05) compared to strained VSMC without anti-VEGF (Figure 4C). Lower concentrations of anti-VEGF (0.004 and 0.04 ug/ml) were tested, but did not result in significant effects (results not shown). Importantly, anti-VEGF also did not significantly affect proliferation or protein expression in non-strained control cells.

There were no differences in proliferation among VSMC incubated with extracellular calcium, with calcium plus the SACC inhibitor GdCl_3_, or in the absence of extracellular calcium (osmotically controlled with Na+) after 48 hours strain. When compared to non-strain VSMC, proliferation among all strain groups were significantly decreased (Table 1).

Antibody protein microarrays of VSMC lysates revealed 46 cellular proteins and/or protein phosphorylation states that changed ± 25% from control (Table 2). Of interest, MAPK cascade peptides including MEK1, MEK2, phospo-MEK1^T385, T291, S298^, and phospho-Erk1/2^T202+Y204/T185+T187 ^were universally increased in strained VSMC compared to control. PKCλ/τ, phospo-PKCλ/τ^T555^, PKCζ, PKCθ, phospo-PKCθ^S695^, PKCμ, phospo-PKCη^T655^, PKCε, phospo- PKCδ^S645 ^were also universally elevated compared to control. However, Cdk4, Cdk6, phospo-Cdk1/2^T14+T15^, and Cdk2 were all decreased in cell lysates of strained versus control (Table 2).

**Table 2:** Effects of strain on VSMC intracellular signaling.

MEK1/ERK		PKC		CDK	
***Protein***	***%CFC***	***Protein***	***%CFC***	***Protein***	***%CFC***
		
MEK1	+148%	PKC λ/ι	+41.4%	Cdk4	-26%
MEK1^T385^	+68.1%	PKC λ/ι^T555^	+90.3%	Cdk6	-39.3%
MEK1^T291^	+159%	PKC ζ	+87%	Cdk1/2^T14+Y15^	-50%
MEK1^S298^	+62%	PKC θ	+231%	Cdk2	-49.6%
MEK2	+188.1%	PKC θ^S695^	+44.6%		
ERK^T202+Y204/T185+Y187^	+130%	PKC μ	+97.3%		
		PKC ε	+30%		
		PKC δ	+40.5%		

%CFC = % change from control (non-strained) cells; T = threonine phosphorylation at sites stated, S = serine phosphorylation at sites stated. N = 10 pooled samples for each treatment group. *MEK = MAPK/ERK kinase protein-serine kinase; MAPK = mitogen-activated protein-serine kinase; ERK = extracellular regulated protein-serine kinase; PKC = protein-serine kinase; CDK = cyclin-dependent protein-serine kinase*.

## Discussion

Our study shows that cyclic strain (CS) causes an antimitogenic response in VSMC, changes to actin/cell alignment, hypertrophy, and strain-regulated cytokine secretion. Secretion of NO and VEGF, among other cytokines, are up-regulated in strained cells temporally corresponding to the observed decreases in proliferation. While exogenous VEGF alone (in the absence of strain) does not inhibit proliferation, neutralization of VEGF secreted from strained cells is effective in restoring normal proliferative responses in physiologically strained VSMC. These data suggest that VEGF is necessary, but is not sufficient to cause the antiproliferative effects seen with CS. The antimitogenic intracellular signaling mechanisms responsible are extracellular calcium-independent, but potentially mediated by downregulation of Cdks.

VSMC and their major actin stress fibers aligned nearly perpendicular to the dominant strain vector after 48 hours of CS, a response which is consistent with our previous findings [20]. In the *absence *of serum, we found alignment was dependent on strain and autocrine NO signaling [20]. Here we report similar findings in the *presence *of serum, suggesting that VSMC actin alignment is dependent upon CS and NO, but independent of serum-derived growth factors and cytokines present within the media prior to strain. We found VSMC exhibited a hypertrophic response to strain evidenced by significantly increased protein:DNA ratio (Figure 2D) and increases in area and perimeter (Figure 2A-B). These data are consistent with in situ studies which also suggest a hypertrophic response in strained VSMC [21,22].

CS causes an antimitogenic response in VSMC's (Figure 2D and 4C). While we take into account VSMC in a uniculture-only model, such a response in vivo would cause leaky blood vessels with poor VSMC coverage [23]. In vivo, VSMC proliferation is likely influenced by other factors such as PDGF and IGF-1 released by endothelial cells [24] to maintain a growth equilibrium between strain induced anti-mitogenesis and paracrine mediated VSMC proliferation. While we recognize that other cell types can influence VSMC proliferation, for this study we were interested only in soluble mediators secreted by VSMC in response to strain that may self-regulate VSMC proliferation through an autocrine mediated manner.

Autocrine cytokines have been implicated in strain-induced hyperplasia (e.g., VEGF, IGF-1, PDGF, angiotensin II). We and others have found that some autocrines may be alternatively regulated in response to strain duration and magnitude. Quinn et al. has shown that a 15% equiradial strain increased the synthesis of VEGF mRNA, while a 25% equiradial strain had no affect VEGF mRNA [25]. This same group reported that while VEGF mRNA expression increases significantly after 24 hrs of strain, there was no difference when compared to non-strain cells after 48 hours strain [25]. We similarly observed a 1.8 fold increase in VEGF secretion at 18 hours which then leveled to control at 48 hours (Figure 4A). The addition of anti-VEGF polyclonal antibody was sufficient to reverse VSMC antimitogenic response to strain (Figure 4C) suggesting that VEGF is indeed a key mediator in the VEGF antiproliferative response. Others have shown in vitro exogenous VEGF (30 - 100 ng/ml) inhibits VSMC proliferation in static conditions [11,26] possibly though inhibition of MEK1/2 - ERK1/2 phosphorylation. However, exogenous incubation of VEGF at concentrations approximate to secretory levels expressed in our model (0.03 ng/ml) was insufficient to inhibit proliferation in non-strain cultures (Figure 4B). This suggests that strain itself is an important cofactor in VEGF-induced antimitogenesis. Secretions of several other cytokines were upregulated in response to strain with documented roles in mediating both VSMC pro- and anti-mitogenic response; pro-inflammatory cytokines CINC-3, CNTF, Fractalkine, GM-CSF, IL-1β, IL-6, MCP-1, and BETA-NGF; anti-inflammatory cytokines IL-4 and IL-10; VEGF and leptin (Figure 3). The antiproliferative response we observed likely culminates from a balance among both pro- and anti-mitogenic cytokines working alongside VEGF. Exploring whether antimitogenesis is due to strain per se or resultant autocrine growth factors/cytokines in our experiments is to be determined.

Intracellular transmission of mechanical strain stimuli that induce VSMC mitogenic responses are highly dependent on the mitogen activated protein kinase (MAPK) cascade [1]. Mechanical strain is known to activate all three members of the MAP kinase cascade: ERK1/2, JNKs, p38 MAPK [27,28]. Our results indicate that neither the JNK nor p38 MAPK were elevated during strain, suggesting these two signaling cascades were not activated by our strain paradigm (results not shown). However, our results did show ERK1/2 signaling cascade activation through MEK phosphorylation at threonines 385 and 291, and serine 298. These sites have been previously shown to induce MEK activation [29]. MEK1/2 then activate downstream ERK1/2 by phosphorylating ERK threonines 202 and 185 and ERK tyrosines 204 and 187 [30-32]. This is consistent with our findings of increased ERK1/2 phosphorylation at these sites (Table 2). Furthermore, protein kinase C (PKC) is also a key upstream regulator of ERK1/2 [33] which was also found to be upregulated in response to strain. This study shows 48 hours of CS activates PKC θ and δ by phosphorylation at serine 695 and serine 645 respectively and activates [34] atypical PKC λ/ι by phosphorylation of threonine 555 (Table 1). Although MAPK and PKC play important roles in strain-induced VSMC proliferation, the outcome of these signaling cascades, and thus progression through the cell cycle, is ultimately regulated by cyclin-Cdk complexes inside the cell nucleus which may be regulated in part by NO.

NO is a key mediator of the antimitogenic response which down regulates the expression of several Cdks [35]. Our previous studies have shown that NO is activated through the inducible nitric oxide synthase (iNOS) pathway during CS in VSMC cultured without serum [20]. Our latest results show NO production is significantly elevated vs. control (71%) after 48 hours of strain in the presence of serum (Table 1). This suggests that NO increases are strain-dependent and serum factor-independent. While our observed decrease in VSMC proliferation appears to be partially attributed to VEGF (Figure 4C), co-regulation by iNOS may also play a central role in strain-induced anti-proliferative response of VSMC.

Proliferation requires the expression of cyclin dependent kinases (Cdks) and our results show decreased expression of Cdk2, Cdk4, and Cdk6 after 48 hours of strain (Table 2). Decreased proliferation due to decreased expression of Cdk has been identified for VSMC suggesting that strain may be acting through a Cdk inhibitory pathway. NO-mediated downregulation of Cdk2 activity and cyclin A gene transcription [35] may help to explain our results which show a decrease in strain-induced VSMC proliferation despite MAPK and ERK1/2 upregulation. Decreased expression of both Cdk4 and Cdk6 also suggests attenuated numbers of cyclin D/Cdk4, Cdk6 complexes, and hence a slowing of VSMC movement past the G_0/1 _restriction point. Our results also showed a decrease in the phosphorylation of Cdk1/2 at threonine 14 and tyrosine 15 (Table 2). These two phosphorylation domains are critical for the inhibition of cyclin/Cdk complexes [36] suggesting a lack of proliferative inhibition via this mechanism. However, the down-regulation of Cdks 2, 4, and 6 in response to strain appears to override the mitogenic effect of decreased phosphorylation of Cdk1/2 resulting in a decrease in VSMC proliferation.

## Conflict of interests

The authors declare that they have no competing interests.

## Authors' contributions

JS was the leading technician of this project and was involved in all area of experimentation, statistical analysis and drafting the manuscript. KM participated in designing the in vitro biomechanical strain paradigm and assisted in the protein cytokine array testing. DB conducted the calcium channel blocker studies. MH and TC were involved with statistical analysis, data interpretation and drafting the manuscript. As the principle investigator PS conceived of the study, and participated in its design and coordination and helped to finalize the manuscript. All authors read and approved the final manuscript.
